# The Regulatory Roles of Non-coding RNAs in Angiogenesis and Neovascularization From an Epigenetic Perspective

**DOI:** 10.3389/fonc.2019.01091

**Published:** 2019-10-24

**Authors:** Itzel Alejandra Hernández-Romero, Lissania Guerra-Calderas, Marisol Salgado-Albarrán, Tatiana Maldonado-Huerta, Ernesto Soto-Reyes

**Affiliations:** Natural Sciences Department, Universidad Autónoma Metropolitana-Cuajimalpa, Mexico City, Mexico

**Keywords:** angiogenesis, non-coding RNA, epigenetics, neovascularization, next generation sequencing, miRNAs, lncRNAs, circRNA

## Abstract

Angiogenesis is a crucial process for organ morphogenesis and growth during development, and it is especially relevant during the repair of wounded tissue in adults. It is coordinated by an equilibrium of pro- and anti-angiogenic factors; nevertheless, when affected, it promotes several diseases. Lately, a growing body of evidence is indicating that non-coding RNAs (ncRNAs), such as miRNAs, circRNAs, and lncRNAs, play critical roles in angiogenesis. These ncRNAs can act *in cis* or *trans* and alter gene transcription by several mechanisms including epigenetic processes. In the following pages, we will discuss the functions of ncRNAs in the regulation of angiogenesis and neovascularization, both in normal and disease contexts, from an epigenetic perspective. Additionally, we will describe the contribution of Next-Generation Sequencing (NGS) techniques to the discovery and understanding of the role of ncRNAs in angiogenesis.

## Introduction

In the vascular network, blood vessels act as channels for nutrients, oxygen delivery, and metabolic waste evacuation. The growth of new capillary vessels, known as angiogenesis, plays key roles in embryonic development and in tissue homeostasis and remodeling in adults, as well as in cancer initiation and progression (1, 2). The balance between pro- and anti-angiogenic factors (such as VEGF, PDGF, and TSP-1/2) coordinates angiogenesis and other neovascularization mechanisms such as intussusceptive angiogenesis, vasculogenesis, lymphangiogenesis, vessel co-option, and vasculogenic mimicry (3–5).

Over the last few decades, the study of angiogenesis has helped researchers to understand vascular physiology and its implications for several diseases. For instance, in atherosclerosis, ischemia, and retinopathy, excessive or insufficient vascular growth can affect the behavior of endothelial and smooth muscle cells (6, 7). Studies of the neovascularization processes have also provided molecular targets for the development of therapies to delay cancer progression, since it is well-known that angiogenesis is an essential process that is altered in tumors (8).

Nowadays, the study of the molecular mechanisms involved in angiogenesis is being built on different experimental approaches, such as cell migration, proliferation, and metabolic assays or histological and tri-dimensional models, that approach specific stages of angiogenesis; however, only pieces of the puzzle have been elucidated (9). With advances in high-throughput genomic technologies such as microarrays, next-generation sequencing (NGS), and bioinformatic analyses, a genome-wide perspective of the elements involved in the angiogenic process is now being taken. Some of the newest players revealed by these approaches are non-coding RNAs (ncRNAs), which have gained relevance in the field of epigenetics (10–12). Therefore, in this review, we will describe the epigenetic regulatory functions of ncRNAs in physiological angiogenesis and vascular diseases, as well as the contribution of NGS technologies to the discovery of new roles for ncRNAs that are associated with angiogenesis.

## An overview of epigenetics

In 1939, the term “epigenetics” was coined by Conrad Hal Waddington (13). Today, one of the most accepted definitions of the term explains that “epigenetics is the study of the heritable changes in gene expression that cannot be explained by alterations in the DNA sequence” (14). Among the epigenetic components that coordinate nucleus organization and gene transcription are DNA methylation, histone post-translational modifications (PTMs), and histone positioning, but recently, ncRNAs have been incorporated as epigenetic modifiers, because many of these can function as scaffolding elements to transport proteins with epigenetic functions (15). Each of these processes is stimulated by the signals derived from a dynamic epigenetic code that is established on the chromatin depending on the physiological and extracellular context. The writers, readers, and erasers of this code are proteins that place, recognize, or remove chemical modifications of DNA nucleotides and within the amino-terminal regions of histones. Most chromatin “writers” are methyltransferases that catalyze the transfer of methyl groups. DNA methylation occurs predominantly in regions enriched in CpG sites. The occurrence of methylation at the promoter regions of genes is associated with gene silencing. PTMs alter the regulation of gene transcription by changing the structure of chromatin depending on the particular residue that is modified (16, 17). The “readers” are proteins that recognize and associate with the epigenetic modifications, interpret them, and, in many cases, promote the assembly of protein complexes. The erasers remove the modifications and, therefore, alter signaling components that contribute to the regulation of gene expression. Recently, it has been reported that ncRNAs can mediate the binding of epigenetic proteins to their target sequences. Though they do not function alone as “classic” epigenetic modifiers, they play a vital role in both the recruitment and transcriptional regulation of epigenetic modifiers (18). In fact, multiple chromatin-remodeling enzymes have been shown to directly contact ncRNAs, including Enhancer of Zeste Homolog 2 (EZH2) and Suppressor of Zeste 12 Protein Homolog (SUZ12) (writer and eraser within the Polycomb repressive complex 2/PRC2, respectively), and nuclear architectural proteins like Yin Yang 1 and CTCF, among others (19–22). The incorporation of ncRNAs as epigenetic elements has opened up new fields of study in which they have been shown to regulate gene expression. In the following pages, we will provide an overview of the ncRNAs involved in angiogenesis, focusing on those involved in epigenetic processes.

## MiRNAs and their epigenetic targets in neovascularization and angiogenic processes

MicroRNAs (miRNAs) are short ncRNAs with a length of 19–23 nucleotides that are conserved in animals, plants, and some viruses (23–25). MiRNAs are transcribed as long pri-microRNAs (pri-miRNA) and are subsequently processed to ~70-nucleotide precursor hairpins (pre-miRNA) by the RNase Drosha (26). Pre-miRNAs are then exported to the cytoplasm and recognized by the RNase DICER, which removes the loop linking the 3′ and 5′ ends of the hairpin, producing a ~20-nucleotide miRNA duplex (27). Later, one of these strands is fused into the RNA Induced Silencing Complex (RISC), where both the miRNA and its messenger RNA (mRNA) target interact (28).

MiRNAs have two main functions: post-transcriptional gene regulation and RNA silencing. They act by pairing bases with a complementary sequence located in the 3′UTR region of target mRNA (29, 30). Consequently, these mRNAs are regulated by one or more mechanisms that include the inhibition of mRNA translation to proteins by ribosomes and by mRNA strand cleavage into two fragments and poly(A) tail shortening that results in mRNA disruption (29, 31). In the last 10 years, the field of miRNA biology has ignited, revealing amazing functions in angiogenesis. These miRNAs have been termed angiomiRs, and they target key angiogenesis molecular drivers, such as metalloproteinases, hypoxia inducible factor 1 (HIF1), cytokines, and growth factors, such as EGFL7, FGF11, PDGFRB, and the vascular endothelial growth factor (VEGF) family (32–34).

MiRNAs are not considered epigenetic components, but some of them are modulated by epigenetic mechanisms. This mainly affects their regulatory region through the incorporation of DNA methylation, repressive histone marks, or the loss of transcriptional factors, as has been reported for *miR-125b1* and *miR-124* (35, 36). Others, known as Epi-miRNAs, can also regulate the gene expression of epigenetic elements, DNA methyltransferases (*DNMTs*) (such as *miR-152, miR-30*, and *miR-148a/b*), histone deacetylases (*HDACs*) (such as *miR-140, miR-1*, and *miR-449a*), and the Polycomb Group of genes (such as *miR-101* and *miR-26a*) (37–44), and some of them have been considered angiomiRs (39, 40). MiRNAs and their identified epigenetic targets in angiogenesis are listed in Table 1.

**Table 1:** Summary of MicroRNAs and their epigenetic targets in angiogenesis and vascular disease.

**Common name**	**Function**	**Mechanism**	**Model or disease**	**References**
MiR-30a-3p	Required for endothelial cell migration during sprouting angiogenesis	Base-pairing with matching sequences within *MeCP2* mRNA	Human umbilical vein endothelial cells	(45)
MiR-101	Inhibitis celular proliferation, migration, invasión and atenuates formation of capillary-like structures	Base-pairing with matching sequences within *EZH2* mRNA	Human brain microvascular endothelial cells and glioblastoma	(46, 47)
MiR20-a	Inhibits angiogenic and hypertrophic programs	Base-pairing with matching sequences within *p300* mRNA	Mouse myocardium	(48)
MiR-137	Inhibits celular proliferation and angiogenesis	Base-pairing with matching sequences within *EZH2* mRNA	Glioblastoma and xenografts of severe combined immunodeficiency mice	(49)
MiR-124	Inhibitis celular proliferation, migration, invasión and formation of capillary-like structures	Base-pairing with matching sequences within *UHRF1* mRNA	Bladder cancer	(44)
MiR-214	Promote angiogenesis and endothelial differentiation	Base-pairing with matching sequences within EZH2 mRNA	Embryonic stem cells	(50)
MiR-200b	Inhibits the formation of capillary-like structures	Posibly base-pairing with matching sequences within p300 mRNA	Diabetic retinopathy	(51)

### MiR-30a-3p

**Figure 1:**
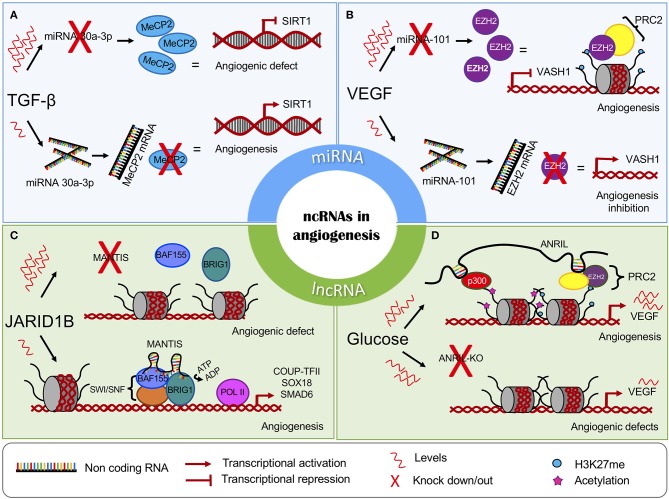
The chromatin regulatory role of non-coding RNAs in angiogenesis. The miRNAs can interfere with the expression of key epigenetic players, leading to the **(A)** induction or **(B)** inhibition of angiogenesis. The lncRNAs regulate **(C)** the activity and **(D)** recruitment of chromatin-modifying complexes. MiRNAs are represented in blue panels, and lncRNAs are represented in green.

Transforming Growth Factor (TGF-β) is a relevant cytokine that functions in the process of vascular homeostasis and is involved in the vascular development of endothelial cells. It has been reported that the administration of TGF-β to endothelial cells leads to decreased *miR-30a-3p* expression. The absence of this microRNA results in increased levels of methyl-CpG-binding protein 2 (MeCP2), a protein associated with silencing of *SIRT1* (45). SIRT1 is necessary for the migration of endothelial cells to occur throughout sprouting angiogenesis, and the loss of this enzyme induces abnormal angiogenesis *in vivo* (52). Conversely, increased levels of *miR-30a-3p* expression lead to the activation of SIRT1 expression (Figure 1A). Further experiments revealed that MeCP2 enhanced the methylation status of the *SIRT1* promoter, probably by DNMT1 recruitment, leading to a reduction in SIRT1 expression and endothelial angiogenic defects (53).

### MiR-101

The microRNA *miR-101* acts as a tumor suppressor, promoting apoptosis and inhibiting cell proliferation, angiogenesis, invasion, and metastasis. *MiR-101* performs its regulatory functions by targeting an abundant range of epigenetic molecular effectors, such as *DNMT3A, EZH2*, and *HDAC9* (54, 55). In endothelial cells, high levels of VEGF are associated with the downregulation of *miR-101*, allowing an increase in *EZH2* (46). EZH2 is associated with the formation of heterochromatin and can affect multiple target genes such as Vasohibin 1 (*VASH1*), which functions as a negative feedback modulator of angiogenesis in vascular endothelial cells (56, 57) (Figure 1B). The overexpression *miR-101* leads to EZH2 repression and the activation of *VASH1* transcription. This evidence, taken together, suggests that *miR-101* is involved in multiple processes such as cellular growth attenuation, migration, and invasion mechanisms and the ability of endothelial cells to form capillary-like structures in glioblastomas (47).

### MiR-20a

*MiR-20a* belongs to the *miR-17-92* cluster and has been linked to breast cancer cells with a high angiogenic profile. High levels of m*iR-20a* are correlated with complex vascular structures and larger vessels, suggesting that *miR-20a* could be used as a potential new angiogenic target (58). Additionally, overexpression of *miR-20a* affects the mRNA stability of the lysine acetyltransferase, p300. In mouse myocardium cells, p300 is a key factor that regulates angiogenic and hypertrophic programs, influencing the expression of many related genes, such as *Hif,1 Vegfc, Vegfa, Angpt1*, and *Egln3*. Interestingly, high p300 levels induce an increase in the expression of *miR-20a*, providing a feedback inhibition loop for p300 that prevents its pro-angiogenic effects (48).

### MiR-137

*MiR-137* has a tumor suppressor gene function that has been reported for several neoplasms (49, 59, 60). It was also reported that this miRNA can inhibit angiogenesis and cell proliferation by EZH2 downregulation in glioblastomas. Overexpression of *miR-137* reduces the mRNA and protein levels of EZH2, while downregulation of *miR-137* is associated with poor prognosis in affected patients (49).

### MiR-124

The miRNA *miR-124* is highly conserved, from nematodes to humans. Three human genes encoding *miR-124* have previously been characterized (*miR124a-1, miR-124a-2*, and *miR-124a-3*) and the majority have been shown to be deregulated in neoplasms (61). Also, it has been shown that expression of *miR-124* is elevated after treatment with certain drugs such as niclosamide. In this case, it is associated with the inhibition of vasculogenic mimicry formation, particularly by reducing levels of phosphorylated STAT3 (62).

Some reports propose that *miR-124* suppresses the E3 ubiquitin ligase with PHD and RING finger domain 1 (*UHRF1*) expression, a factor involved in the recruitment of epigenetic components in bladder cancer tissues. Also, UHRF1 is known to enhance malignancy, inducing cellular proliferation, migration, and angiogenesis (63). *MiR-124* overexpression resulted in UHRF1 suppression through the competitive binding of its 3'-UTR region. In addition, *miR-124* overexpression attenuated tumor growth and cell proliferation *in vivo* and invasion, migration, and vasculogenic mimicry *in vitro*. Further, it reduced VEGF protein levels and levels of the matrix metalloproteinases MMP-2 and MMP-9. A matrigel assay in a three-dimensional culture revealed reductions in tubular channel formation when *miR-124* was over-expressed in bladder cancer cell lines compared to the control group, suggesting that *miR-124* indirectly regulates vasculogenic mimicry in bladder cancer (44).

### MiR-214

Originating from intron 14 of the Dynamina-3 gene (*DNM3*), the primary transcript of *miR-214* produces four different miRNAs (*miR-199-3p, miR-199-5p, miR214-3p*, and *miR-214-5p*) (64). During the endothelial differentiation of embryonic stem cells, the Brain-Derived Neurotrophic Factor (BDNF) promotes angiogenesis, *in vitro* and *in vivo*, by increasing levels of *miR-214*. The *miR-214* inhibits *EZH2* at the post-transcriptional level, leading to reductions in EZH2 occupancy at the *NOS3* promoter (50). Also, *miR-214* controls the BDNF-mediated upregulation of neuropilin 1, VEGF-R, and Crk-associated substrate kinase (50, 65). Thus, *miR-214* is a downstream player within the BDNF signaling pathway that regulates important angiogenic targets.

### MiR-200b

*miR-200b* is part of the miR-200 family, which is organized into two main groups according to seed sequence. The miRNAs of group A are *miR-141* and *miR*−*200a*, while the miRNAs in group B are *miR-200b, miR*−*200c*, and *miR*−*429* (66). Particularly, *miR-200b* has been indicated to have a role in the process of angiogenesis. Studies of malignant neoplasms demonstrated that *miR-200b* controls the epithelial to mesenchymal transition by downregulating p300 (67–70). In addition, p300 activates *HIF1*, which is a transcriptional regulator of *VEGF-A*, and triggers the development of abundant blood vessels (71–73). Since *miR-200b* negatively regulates p300, this miRNA has antiangiogenic properties (51).

In sum, these studies suggest that miRNAs have the capacity to indirectly affect epigenetic pathways in endothelial cells and influence the angiogenic response. This opens up the possibility of considering miRNAs as therapeutic targets or biomarkers, an exciting prospect since therapies for both vascular diseases and cancer are needed. In several diseases, miRNAs have proven to be excellent biomarkers as a result of their high circulating levels. Indeed, analysis of oncogenic and suppressor miRNAs that are found in primary tumors against non-neoplastic cells revealed exosome-mediated sorting mechanisms related to cancer progression (74, 75). It is unknown whether similar mechanisms could be utilized by Epi-miRNAs during the evolution of vascular diseases. Recently, the attention of the scientific community has been focused on other, widely-studied ncRNAs known as long non-coding RNAs (lncRNAs), which have master regulatory functions in angiogenesis.

## Long non-coding RNAs as scaffolds for epigenetic partners in neovascularization

LncRNAs are all ncRNAs larger than 200 nucleotides and are classified according to their proximity to protein-coding genes as intergenic, intronic, bidirectional, sense, and antisense lncRNAs. Massive analyses have revealed that lncRNAs are originated using the same mechanisms as protein-coding genes; however, contrary to protein-coding genes, lncRNAs show a preference for having two-exon transcripts, and most of them lack any protein coding-potential. Also, lncRNAs show tissue-specific expression patterns and are predominantly located in the nucleus rather than the cytoplasm. In fact, there are several lines of evidence that suggest that lncRNAs are significantly more enriched in chromatin than miRNAs (76).

LncRNAs can indirectly modulate DNA methylation at CpG sites, which in turn regulates gene transcription. For example, Tsix recruits DNMT3a to methylate and silence the *XIST* promoter. XIST is an important effector involved in the inactivation of the X chromosome (77). Likewise, the lncRNA Kcnq1ot1 recruits the *de novo* DNA demethylase DNMT1 to control the methylation status of ubiquitously imprinted genes during mouse development (78). LncRNAs can act as guides or scaffolds, facilitating interaction between several proteins, such as those that are part of chromatin-modifying complexes, causing gene activation or repression, depending on the interaction partners involved (79, 80). The polycomb repressive complexes PRC1 and PRC2, the transcriptional repressor element-1 silencing transcription factor REST, its cofactor (REST/CoREST), other epigenetic components like the mixed lineage leukemia protein and the H3K9 methyltransferase G9a, physically interact with lncRNAs (78, 80, 81). In addition, many lncRNAs such as HOTAIR, Xist, Kcnq1ot1, and Breaveheart interact with PRC2, implying that these ncRNAs play a role in recruiting this complex through its subunits (EZH2, SUZ12, EED, RBBP4, and AEBP2) or through a bridging protein (such as JARID2) to their target genes (82, 83). Likewise, the expression of many angiogenesis-related genes involved in the VEGF signaling pathway is regulated through lncRNAs (such as H19, MEG3, and HOTAIR), and recently, researchers discovered that some of them perform their regulatory function by influencing the expression and activity of several epigenetic modulators (20, 22). LncRNAs and their identified epigenetic targets in angiogenesis are listed in Table 2.

**Table 2:** Summary of lncRNAs and their epigenetic targets in angiogenesis and vascular disease.

**Common name**	**Function**	**Mechanism**	**Model or disease**	**References**
MANTIS	Promotes angiogenic sprouting and proper endothelial function	Interplay with BRG1 and favors ATPase activity in chromatin remodeling	Human umbilical vein endothelial cells, glioblastoma, atherosclerosis, and idiopathic pulmonary, arterial hypertension	(80)
ANRIL	Induces the formation of capillary-like structures	Recruitment of EZH2 and p300 to the VEGF promoter	Diabetic retinopathy	(84)
GATA6-AS	Promotes angiogenic sprouting	Binds to LOXL2 and regulates H3K4 trimethylation of angiogenesis- and hypoxia-related genes	Human endothelial cell-based xenograft model	(85)

### MANTIS

MANTIS is a recently discovered lncRNA required for endothelial cell function and proper angiogenesis. MANTIS is induced in the endothelium of glioblastoma tumors and is overexpressed during vascular regeneration in atherosclerosis regression. It alters angiogenic sprouting, tube formation, and epithelial cell migration. Loss of *MANTIS* expression is reported during pulmonary arterial hypertension, and its downregulation also led to the reduced expression of many angiogenesis-related mRNAs (80).

In endothelial cells, *MANTIS* is upregulated following the knockdown of the histone demethylase JARID1B. JARID1B loss triggers increased H3K4me3 levels at transcription start sites (TSS) of the *MANTIS* gene, facilitating gene expression. Interestingly, in patients with idiopathic pulmonary arterial hypertension, a disease characterized by endothelial dysfunction, *MANTIS* expression is downregulated, while JARID1B is upregulated (80).

Novel studies have revealed that MANTIS functions as a scaffold and regulates the activity of Brahma related gene-1 (BRG1), the catalytic subunit of the SWI/SNF chromatin remodeling complex. The MANTIS-BRG1 interaction allows for increased binding of BAF155, which is a core component of the SWI/SNF complex, enhancing BRG1 ATPase activity and chromatin relaxation at the TSS of the transcription factor *COUP-TFII*, which, in turn, recruits RNA Pol II binding and transcription of the pro-angiogenic genes *SOX18* and *SMAD6*. The knockdown of MANTIS reduces BRG1 ATPase activity (80) (Figure 1C).

### ANRIL

ANRIL is an antisense lncRNA from the INK4 locus. It encodes two cyclin-dependent kinase inhibitors, p15 (INK4b) and p16 (INK4a), and a protein known as ARF. All of the genes cooperate in tumor suppressor networks. When these genes are silenced, proatherosclerotic cellular mechanisms are enhanced, such as increased adhesion and diminished apoptosis (86). In fact, ANRIL expression is correlated with the risk of some vascular diseases such as coronary atherosclerosis and carotid arteriosclerosis (87).

It has been shown that ANRIL recruits PRC2 or PRC1 to different target genes by directly interacting with their subunits EZH2, SUZ12, and CBX7 (86, 88, 89). In a diabetic retinopathy cellular model, high glucose levels upregulated ANRIL and VEGF expression. In turn, ANRIL positively regulated EZH2, EED, and p300 levels. Furthermore, ANRIL recruits EZH2 and histone acetyl-transferase p300 to the *VEGF* promoter, enhancing its expression and angiogenic effects. It was shown that ANRIL silencing prevented the formation of capillary-like structures in spite of the angiogenic influence of high glucose levels (84) (Figure 1D). Moreover, ANRIL silencing also promoted *miR-200b* expression, a previously described miRNA that has been shown to be involved in regulating VEGF (90).

### GATA6-AS

GATA6-AS is the hypoxia-regulated long non-coding antisense transcript of *GATA6* and promotes angiogenesis by negatively regulating lysyl oxidase-like 2 (*LOXL2*). LOXL2 catalyzes the oxidative deamination of lysines and hydroxylysines, which results in the generation of non-methylated H3K4 and gene silencing. Thus, GATA6-AS silencing leads to increased LOXL2 activity and transcriptional repression. In the nucleus, the physical interaction between GATA6-AS and LOXL2 positively regulates the expression of several angiogenesis- and hypoxia-related genes, such as periostin and cyclooxygenase-2. It has been shown that GATA6-AS silencing in epithelial cells significantly prevented TGF-β2-induced endothelial to mesenchymal transition and augmented angiogenic sprouting in xenograft models *in vivo* (85).

Like epi-miRNAs, the epi-lncRNAs are excellent candidates biomarkers due to their easy collection and tissue specificity. Although there are few examples of epi-lncRNAs in angiogenesis, the implications behind these interactions provide an interesting view of the mechanisms in which lncRNAs regulate not only the recruitment but also the activity of chromatin modifiers. Another layer of complexity is added if we consider that lncRNAs have many alternative splice forms, including the non-linear, circular RNAs (circRNAs).

## Circular RNAs in neovascularization

Circular RNAs (circRNA) are single-stranded RNAs that are widely conserved in all life domains and form a covalent closed loop (91). The discovery of this type of RNA has occurred fairly recently, and before their discovery, the RNAs were considered the result of errors within the process of gene transcription. These circRNAs are produced by a back-splicing process of pre-mRNA, in which a downstream splice donor is linked to an upstream acceptor (92, 93). The splice forms can circularize from exonic, intronic, or a combination of both regions (EIciRNAs) (94).

In cancer-derived cell lines, it has been reported that changes in DNMTs and the hypermethylation of the CpG islands of some genes that host circRNA can induce gene silencing of both linear RNA and circRNA, suggesting an epigenetic mechanism that produces two molecular “hits” (95). Because circRNA lack 5′ and 3′ ends, these cannot be degraded by exoribonucleases. Instead, circRNA levels may be regulated by endonucleases and exosomal deportation (96). These molecules are stable, abundant and specific to certain cell types, having distinct transcriptional patterns for specific tissues and multiple isoforms in eukaryotic cells (97). CircRNAs have been linked to different biological processes, including cell proliferation, senescence, and apoptosis, among others. The study of circRNA has increased in recent years, since they have been shown to be related to both physiological and pathological processes (98). In fact, circRNAs have been proposed as potential biomarkers for neurological disorders, infectious diseases, cancer, and preeclampsia as a result of their availability in circulating body fluids (99–102).

The circRNAs have transcriptional and post-transcriptional regulatory functions. EIciRNAs such as EIF3J associate with ribonucleoproteins like U1 and the Pol II at the promoters of their parental genes to enhance their own expression (94). Similar to EIciRNAs, some circRNAs (such as ciANKRD52) can positively regulate their own expression through interaction with the Pol II complex (103). Other circRNAs regulate alternative splicing or serve as sponges to bind, store, or sequester miRNAs and other protein complexes containing transcription factors and RNA binding proteins (94, 104, 105). Due to the ability of cirRNA to bind to miRNAs, they have been referred to as miRNA sponges (106). Despite their recent discovery, some evidence suggests that circRNAs are implicated in angiogenesis (e.g., circRNA-MYLK) and many cardiovascular diseases, such as atherosclerosis (e.g., circR-284), myocardial infarction (e.g., ciRS-7), and coronary artery disease (CAD) (e.g., circ_0124644), among others (107, 108). However, to our knowledge, no study has shown that circRNAs have an epigenetic regulatory role in angiogenesis. Similar to the lncRNA ANRIL, a circularized and anti-sense splice variant of the INK4/ARF locus (cANRIL) has been associated with atherosclerotic vascular disease (109). Moreover, in the cytoplasm, the binding of circANRIL to the rRNA-processing machinery impairs its function and causes nucleolar fragmentation and stress signaling (110). These findings suggest that, just like their longer-sized isoform, the variant cANRIL may have a role in the epigenetic regulation of vascular disease.

The study of ncRNA has opened up a new research field, and this has been extended to the genome scale. This type of experimental approach has become common practice in both the research laboratory and at the clinical level. Therefore, along with a growing array of genomic analysis machinery, bioinformatics platforms have also been developed, thus generating a new set of tools for the study and analysis of ncRNA.

## Contribution of NGS technologies to the discovery of new ncRNAs

In recent years, increasing quantities of data have been obtained from NGS technologies such as mass RNA sequencing (RNA-seq), small RNA-seq (smRNA-seq), and single-cell RNA-seq, among others. These technologies have revealed that the human genome encodes for more than 90,000 non-coding RNAs and that these play an important role in several diseases (111). Using publicly available genomic information, it is now possible to discover and characterize novel disease-associated ncRNAs. In the next section, we will describe some of the key discoveries that have been made thanks to NGS data, in which ncRNAs are shown to have roles in angiogenesis and neovascularization processes.

The study of the ncRNAs involved in molecular processes associated with neovascularization and angiogenesis in several diseases can be carried out by using RNA-seq approaches, especially where angiogenesis or neovascularization is one of the causes, risk factors, or consequences of the disorders. Some of the diseases studied in this manner have been ischemia stroke, CAD, hemangioma, and heart failure (HF). Furthermore, angiogenesis and neovascularization are strongly related to endothelial functioning and the transcriptional programming of endothelial progenitor cells (EPCs). Thus, the study of the molecular mechanisms involved in the regulation of EPCs is of great interest. Nevertheless, only a few studies have been conducted on human umbilical vein endothelial cells (HUVEC) or other endothelial models to understand the role of ncRNAs using NGS technologies. In this section, we will provide a compilation of some studies aiming to identify or characterize ncRNAs involved in vascular processes.

First, in 2012 Cheng et al. performed smRNA-seq on umbilical cord blood EPCs (UC-EPCs), which was known for its enrichment in EPCs, and compared the expression profiles against EPCs derived from peripheral blood in adults (PB-EPCs) to understand the underlying mechanisms involved the functional differences between these two models. They identified specific patterns of miRNAs (miRNome) in UC-EPCs and PB-EPCs in which 54 miRNAs were overexpressed in UC-EPC and 50 miRNAs were overexpressed in PB-EPCs. For instance, UC-EPCs expressed miRNAs involved in angiogenesis such as *miR-31* and *mir-18a*, while PB-EPCs are enriched in tumor-suppressive miRNA expression such as that of *miR-10a* and *mir-26a* (112).

A study performed by Wang and colleagues in 2014 revealed that there was cooperation between VEGF and miRNAs in CAD progression. They performed smRNA-seq and identified EPC-specific miRNome that was related to angiogenic processes, which suggests that miRNAs in EPCs with a poor capacity to enhance angiogenesis might have higher levels of miRNAs targeting VEGF. Indeed, they identified anti-VEGF miRNAs such as *miR-361-5p* that were enriched in EPCs and in the plasma of patients with CAD (113).

Also, atherosclerosis appears to be one of the factors leading to CAD. In 2018, Mao and colleagues conducted a study to identify miRNAs linked with carotid atherosclerosis. They performed a differential expression analysis to identify genes that were specifically associated with either primary or advanced atherosclerotic plaque tissues. Using public databases, they predicted 23 miRNAs that targeted the differentially expressed genes, such as *miR-126, miR-155, miR-19A*, and *miR-19B*, which can play a regulating role in neovascularization and angiogenesis (114).

Furthermore, a study from Liu et al. (115) identified differentially expressed ncRNAs that were predicted to be involved in the regulation of high-density lipoprotein (HDL) metabolism, the deregulation of which is believed to be one of the main causes of CAD. To this end, they treated HUVEC cells with HDL from healthy subjects and patients with CAD and hypercholesterolemia. After RNA-seq analysis, 41 ncRNAs were identified, and researchers were able to show that the ncRNAs, along with protein-coding genes such as DGKA and UBE2V1, have critical functions in vascular cells (115).

Additionally, it is well-known that endothelial cell metabolism is sensitive to hypoxia, which is an adverse effect of atherosclerotic lesions in humans. In 2018, Moreau et al. investigated the lncRNA profiles of HUVEC cells using global run-on sequencing (GRO-Seq). GRO-seq is a sequencing method that measures active transcription, identifying newly synthetized RNA, and providing sufficient resolution to map the position and orientation of transcripts detected. This group aimed to discover changes in the expression patterns of lncRNAs in HUVEC cells exposed to hypoxia and demonstrated that hypoxia affects the transcription of ~1,800 lncRNAs. Among the most relevant lncRNAs identified were MALAT1, HYMAI, LOC730101, KIAA1656, and LOC339803, which were differentially expressed in human atherosclerotic lesions compared to normal vascular tissue (116).

In contrast, heart and circulatory system diseases often involve changes in vascular smooth muscle or cardiac cells. In 2018, Cheng et al. used RNA-seq to identify circRNAs in human aortic valves. They recognized 1,412 specific circRNAs, most of which originated from exons of their host genes. Furthermore, after performing a gene ontology enrichment analysis, they found that the host genes were associated with pathways regulating aortic valve function (ECM-receptor interaction pathway, ErbB signaling pathway, and vascular smooth muscle contraction pathway) (117). In addition, Bell et al. identified novel lncRNAs in human vascular smooth muscle cells in 2014. This work expanded our knowledge of the relevance of lncRNAs in the control of smooth muscle cells. The researchers performed an RNA-seq experiment examining expression patterns in human coronary artery smooth muscle cells. Their analysis revealed 31 novel lncRNAs. They discovered and characterized a novel vascular cell-enriched lncRNA that they named *SENCR*. They performed RNA-seq after knockdown of *SENCR* and observed that expression of Myocardin and genes involved in the contraction of smooth muscle were reduced, while expression of other promigratory genes was enhanced (118). These results have enhanced our understanding of vascular cells and should be further studied in order to discern lncRNAs in vascular diseases. Finally, in 2015, Di Salvo et al. analyzed the expression profiles of cells derived from 22 human hearts from patients with Heart Failure (HF) vs. non-HF donor hearts. Initially, they discovered 2,085 lncRNAs, and subsequent analyses revealed 48 differentially expressed lncRNAs in HF patients. Among these, AP000783.2, RP11-403B2.6, and RP11-60A24.3 were identified (119).

Angiogenesis and neovascularization processes affect the prognosis of patients who have suffered from brain stroke ischemia. Thus, the identification of ncRNAs involved in these processes might be useful for their further use as drug targets or biomarkers for the disease. Therefore, Zhang et al. (120) aimed to uncover which ncRNAs have altered expression profiles after cerebrovascular dysfunction in ischemic stroke. Using bulk RNA-seq, they profiled lncRNA signatures in primary brain microvascular endothelial cells after oxygen-glucose deficiency. This approach allowed for the identification of 362 differentially expressed lncRNAs. The top three lncRNAs that were upregulated were Snhg12, Malat1, and lnc-OGD 1006, while the top three down-regulated lncRNAs were 281008D09Rik, Peg13, and lnc-OGD 3916 (120).

Another disease model that has been studied in order to identify ncRNAs involved in angiogenesis and neovascularization is infantile hemangioma (IH), which is a type of vascular tumor in infants. Li et al. investigated whether ncRNAs have a role in IH pathogenesis in 2018. The researchers used a bulk RNA-seq approach to examine global ncRNAs expression profiles in IH patients compared to their matched, normal-skin controls. In this study, researchers identified 256 lncRNAs and 142 miRNAs that were differentially expressed. They also found more than a thousand sponge modulators involved in miRNA-, lncRNA-, and mRNA-mediated interactions. These findings suggest the presence of an endogenous ncRNA regulatory network associated with the development of IH and other vascular diseases (121).

Overall, the studies described above have shown that NGS technologies can be very effective in identifying and characterizing ncRNAs. This type of technology has helped researchers to understand the regulatory role of ncRNAs in angiogenic and neovascularization processes. However, studies in this field are just emerging, and additional research will be required to expand our knowledge and translated into clinical use.

## Current approaches used to discover new ncRNAs

**Figure 2:**
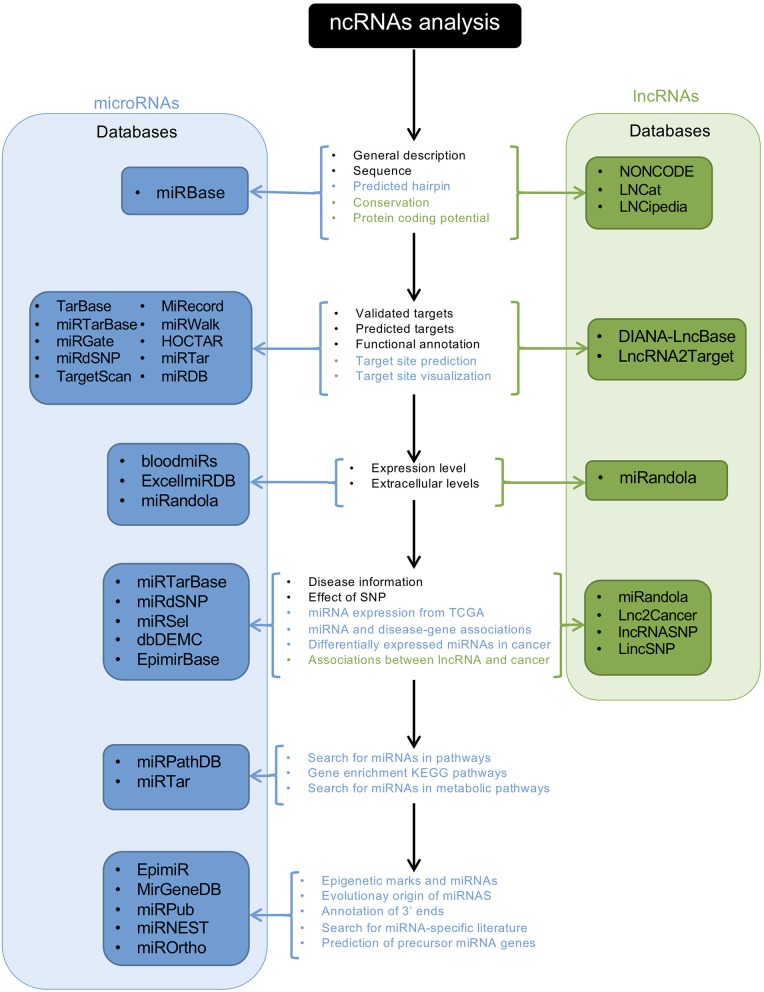
Schematic representation of an ncRNA analysis workflow using databases. The central panel shows the available analyses provided by the databases; letters in black indicate the available analyses for miRNAs and lncRNAs; blue letters indicate analysis for miRNAs, and green letters the analyses for lncRNAs. Blue and green panels show the names of the available databases for miRNAs and lncRNAs, respectively.

After the development of NGS technologies, ncRNAs have been discovered, and multiple efforts have been made to organize, collect, provide, and unify all available information regarding ncRNAs so that it can be accessed by the research community. Furthermore, new methods have developed to predict and identify novel ncRNAs. Here we present some of the cutting-edge bioinformatics approaches currently being used to study ncRNAs and give some examples of how they are used in the study of neovascularization processes (Figure 2). For a detailed explanation, see the following reference (122).

Transcriptome-wide association studies can be performed to identify expression-trait associations where ncRNAs might be involved. This method can identify single-nucleotide polymorphisms (SNPs) located in transcribed regions of ncRNA genes that can be related to a specific phenotype. A second bioinformatic approach is the use of tools for the prediction of primary, secondary, and tertiary ncRNA structures to obtain information about their potential function. This method has been used for circRNAs, smRNAs, and lncRNAs. The third approach to studying ncRNAs is the use of biological networks. These types of analyses enhance our understanding of the function of ncRNAs by integrating expression, regulatory, and protein–protein interaction networks. NcRNAs are highly connected in these networks and can influence more than one target gene in order to produce a specific phenotype. These approaches can identify disease-specific regulatory modules where ncRNAs play an important role (122).

Though the effective methods described above can be used to discover and understand the biological functions of ncRNAs, they have not been adequately exploited to reveal the roles of ncRNAs in angiogenesis or neovascularization. So far, only a few studies have used advanced bioinformatics tools for this purpose. For example, in 2018, Li et al. detected novel circRNAs related with IH using RNA-seq data. The best experimental approach for the detection of circRNAs is the use of deep sequencing of RNA treated with RNase R (which leaves a circRNA-enriched sample). The availability of tools to predict novel circRNAs from RNA-seq data is of great value, given that RNA-seq data are much more highly available (122). Thus, Li et al. used circRNAFinder, a tool able to predict circRNAs from bulk RNA-seq experiments, and identified 249 circRNA candidates differentially expressed between IH and matched normal skin samples. The circRNAs hsa_circRNA001885 and hsa_circRNA006612 where further investigated by this group, providing novel insights about the disease (123).

As shown previously, the development of tools used to predict and identify novel ncRNAs is invaluable. The increasing number of RNA-seq experiments and access to databases will increasingly facilitate the discovery of novel ncRNAs, and the characterization of ncRNAs will become increasingly straightforward. For instance, ANGIOGENES is a database that has been created to store information related to angiogenic processes. It depicts experimental data obtained from RNA-seq experiments in endothelial cells. This allows for the *in-silico* detection of genes expressed in several endothelial cell types from different tissues. ANGIOGENES uses publicly-available RNA-seq experiments and identifies endothelial cell-specific ncRNAs in human, mouse, and zebrafish. The database facilitates further analyses using GO enrichment terms and is available online (124). In addition to ANGIOGENES, EndoDB is another database that retrieves information about endothelial cells from different platforms for several species (125). Other databases are available for the study of ncRNAs; nevertheless, these are not specialized in angiogenesis or neovascular processes. Databases and tools used for the study of ncRNAs are listed in Table 3.

**Table 3:** Databases and tools for the ncRNAs study.

**Database**	**ncRNAs**	**Website**	**Species**	**References**
**ANNOTATION RESOURCES**				
miRbase	microRNAs	http://www.mirbase.org/	All	(126, 127)
NONCODE	lncRNAs	http://www.noncode.org/	All	(111)
LNCipedia	lncRNAs	https://lncipedia.org/	Human	(128)
LNCact	lncRNAs	http://biocc.hrbmu.edu.cn/LNCat/	Human	(129)
**TARGET RESOURCES**				
TarBase	microRNAs	http://diana.imis.athena-innovation.gr/DianaTools/	Human/mouse, fruit fly, worm, and zebrafish	(130)
miRTarBase	microRNAs	http://mirtarbase.mbc.nctu.edu.tw	Human, mouse, virus	(131)
miRGate	microRNAs	http://mirgate.bioinfo.cnio.es	Human, rat, mouse	(132)
miRdSNP	microRNAs	http://mirdsnp.ccr.buffalo.edu	Human	(133)
TargetScan	microRNAs	http://www.targetscan.org/vert_72/	Human	(134)
CSmiRTar	microRNAs	http://cosbi.ee.ncku.edu.tw/CSmiRTar/	Human, mouse	(135)
MiRecords	microRNAs	http://c1.accurascience.com/miRecords/	Huma, rat, mouse, fly, worm, chicken	(136)
miRSel	microRNAs	https://services.bio.ifi.lmu.de/mirsel/	Human, mouse	(137)
miRWalk	microRNAs	http://zmf.umm.uni-heidelberg.de/apps/zmf/mirwalk2/	Human, mouse	(138)
miRPathDB	microRNAs	https://mpd.bioinf.uni-sb.de/	Human, mouse	(139)
HOCTARdb	microRNAs	http://hoctar.tigem.it/	Human	(140)
miRTar	microRNAs	http://mirtar.mbc.nctu.edu.tw/human/	Human	(141)
miRDB	microRNAs	http://www.mirdb.org/	Human, rat, mouse, dog, chicken	(142)
DIANA-LncBase	microRNAs lncRNAs	www.microrna.gr/LncBase	Human, mouse	(143)
LncRNA2Target	lncRNAs	http://123.59.132.21/lncrna2target	Human, mouse	(144)
**CELL TYPE SPECIFIC RESOURCES**				
bloodmiRs	microRNAs	http://134.245.63.235/ikmb-tools/bloodmiRs/	Human	(145)
ExcellmiRDB	microRNAs	http://www.excellmirdb.brfjaisalmer.com/%27%	Human	(146)
miRandola	microRNAs	http://mirandola.iit.cnr.it/	Human	(147)
miREnvironment	microRNAs	http://www.cuilab.cn/miren	Human	(148)
HMED	microRNAs	http://bioinfo.life.hust.edu.cn/smallRNA/	Human	(149)
**DISEASE-RELATED RESOURCES**				
dbDEMC	microRNAs	http://www.picb.ac.cn/dbDEMC/	Human	(150)
EpimiRBase	microRNAs	https://www.epimirbase.eu/	Human	(151)
HMDD	microRNAs	http://www.cuilab.cn/hmdd	Human	(152)
OncomiRDB	microRNAs	http://lifeome.net/database/oncomirdb/	Human	(153)
LncRNADisease	lncRNAs	http://www.cuilab.cn/lncrnadisease	Human	(154)
Lnc2Cancer	lncRNAs	http://www.bio-bigdata.com/lnc2cancer/	Human	(155)
lncRNASNP	lncRNAs	http://bioinfo.life.hust.edu.cn/lncRNASNP/	Human	(156)
LincSNP	lncRNAs	http://bioinfo.hrbmu.edu.cn/LincSNP/	Human	(157)
**OTHER RESOURCES**				
EpimiR	microRNAs	http://210.46.85.180:8080/EpimiR/	Human	(158)
MirGeneDB	microRNAs	http://mirgenedb.org/	All	(25)
miRBaseTracker	microRNAs	http://mirbasetracker.org/	All	(127)
mirPub	microRNAs	http://www.microrna.gr/mirpub/	All	(159)
miRNEST	microRNAs	http://rhesus.amu.edu.pl/mirnest/copy/	All	(160)
miROrtho	microRNAs	http://cegg.unige.ch/mirortho	All	(161)

We know that endothelial cells are heterogeneous; for instance, they function differently depending on vessel type (162). To uncover the molecular mechanisms controlling this heterogeneity, single-cell RNA sequencing analyses (scRNA-seq) have the potential to enhance our understanding of vascular biology. ScRNA-seq is currently being used to study and assess cellular heterogeneity. Particularly with respect to cancer research, this approach has proved to be valuable (163–165); nevertheless, its use in vascular research is just beginning. Recently published studies have mostly focused on protein-coding genes (166, 167). The participation of ncRNAs, along with epigenetic factors, in regulating the metabolic activities of endothelial cells from a single-cell perspective in vascular development and diseases is not yet clear.

## Concluding remarks

ncRNAs comprise a new frontier in genetic regulation that has impacts on several research areas. Undoubtedly, the study of angiogenesis and neovascularization has been enhanced through the integration of the study of ncRNAs and epigenetics. Further, ncRNAs are involved in the regulation of several angiogenic targets through epigenetic mechanisms. On the basis of this relationship, a new field of opportunity has emerged in which biomarkers and specific therapies may be identified that can improve the treatment of different vascular diseases and cancers. NGS platforms allow for the global analysis of ncRNA expression and can be used to compare different physiological and pathological processes. Most of the pathways and mechanisms controlling the ncRNA-mediated regulation of angiogenesis remain unexplored. It is likely that new research strategies implementing an epigenetic perspective will facilitate future discoveries.

## Author Contributions

IH-R, LG-C, MS-A, TM-H, and ES-R wrote the manuscript. MS-A and IH-R did the artwork. TM-H and LG-C combined their information to make the tables. All authors contributed to manuscript revision and read and approved the submitted version.

### Conflict of Interest

The authors declare that the research was conducted in the absence of any commercial or financial relationships that could be construed as a potential conflict of interest.

## Abbreviations

BDNF: Brain-Derived Neurotrophic Factor
BRG1: Brahma related gene-1
CAD: Coronary Artery Disease
circRNA: circular RNAs
DEGs: Differentially Expressed Genes
DNMT: DNA methyltransferase
EIciRNAs: Exon-intron circular RNAs
EPCs: Endothelial Progenitor Cells
EZH2: Enhancer of Zeste Homolog 2
GRO-Seq: Global run-on sequencing
HDAC: Histone deacetylase
HDL: High-density lipoprotein
HF: Heart Failure
HIF1: Hypoxia Inducible Factors 1
HUVEC: Human Umbilical Vein Endothelial Cells
IH: Infantile hemangioma
lincRNAs: intergenic lncRNAs
lncRNAs: long non-coding RNAs
LOXL2: Lysyl oxidase-like 2
MeCP2: Methyl-CpG-binding protein 2
miRNAs: microRNAs
MMP: Matrix metalloproteinase
mRNA: messenger RNA
ncRNAs: non-coding RNAs
NGS: Next-Generation Sequencing
PB-EPCs: Peripheral Blood EPCs
PRC: Polycomb Repressive Complex
pre-miRNA: precursor hairpin miRNA
REST: Repressor Element-1 Silencing Transcription
RNA-seq: RNA sequencing
SIRT1: NAD-dependent deacetylase sirtuin1
smRNA-seq: small RNA-seq
SNPs: Single Nucleotide Polymorphisms
SUZ12: Suppressor of Zeste 12 Protein Homolog 2
TF: Transcription Factor
TGF-β: Transforming Growth Factor
TSS: Transcription Start Sites
UC-EPCs: Umbilical Cord EPCs
UHRF1: E3 ubiquitin ligase with PHD and RING finger domain 1
VASH1: Angiogenesis inhibitor vasohibin 1
VEGF: Vascular Endothelial Growth Factor.
